# Alpha-1 Antitrypsin Prevents the Development of Preeclampsia Through Suppression of Oxidative Stress

**DOI:** 10.3389/fphys.2016.00176

**Published:** 2016-05-24

**Authors:** Yaling Feng, Jianjuan Xu, Qin Zhou, Rong Wang, Nin Liu, Yanqun Wu, Hua Yuan, Haisha Che

**Affiliations:** ^1^Department of Obstetrics and Gynecology, Wuxi Maternal and Child Health Hospital, Nanjing Medical UniversityWuxi, China; ^2^Neonatal Department, Wuxi Maternal and Child Health Hospital, Nanjing Medical UniversityWuxi, China

**Keywords:** alpha-1 antitrypsin, preeclampsia, oxidative stress, p38, PAK

## Abstract

Preeclampsia (PE) and its complications have become the leading cause of maternal and fetal morbidity and mortality in the world. And the development of PE is still barely predictable and thus challenging to prevent and manage clinically. Oxidative stress contributes to the development of the disease. Our previous study demonstrated that exogenous Alpha-1 antitrypsin (AAT) played a cytoprotective role in vascular endothelial cell by suppressing oxidative stress. In this study, we aim to investigate whether AAT contributes to the development of PE, and to identify the mechanism behind these effects. We found that AAT levels were significantly decreased in placenta tissues from women with PE compared that of healthy women. Notably, we demonstrate that AAT injection is able to relieve the high blood pressure and reduce urine protein levels in a dose-dependent manner in PE mice. In addition, our results showed that AAT injection exhibited an anti-oxidative stress role by significantly reducing PE mediated-upregulation of ROS, MMP9 and MDA, and increasing the levels of SOD, eNOS, and GPx with increased dosage of AAT. Furthermore, we found that AAT injection inactivated PE mediated activation of PAK/STAT1/p38 signaling. These findings were confirmed in human samples. In conclusion, our study suggests that exogenous AAT injection increases the antioxidants and suppresses oxidative stress, and subsequent prevention of PE development through inactivation of STAT1/p38 signaling. Thus, AAT would become a potential strategy for PE therapy.

## Introduction

Preeclampsia (PE) and its complications have become the leading cause of maternal and fetal morbidity and mortality in the world, accounting for nearly 40% of births delivered at early gestation (Sanchez-Aranguren et al., 2014). About 5–10% of all pregnant women worldwide present PE, and the development of PE is still barely predictable and thus challenging to prevent and manage clinically (Cindrova-Davies, 2014). PE is characterized by hypertension, proteinuria, and edema. Although the pathophysiology of preeclampsia remains undefined, it is accepted that early in pregnancy, PE is initiated by inadequate trophoblast invasion, which produces an increase in oxidative stress contributing to the development of the disease in the later phases (Hansson et al., 2014).

Oxidative stress of the placenta is a key risk factor by resulting in endothelial dysfunction via direct actions on the vasculature (Sanchez-Aranguren et al., 2014). Several important antioxidants, such as superoxide dismutase (SOD) and glutathione peroxidase (GPx) that protects the vasculature from ROS and maintains the vascular function, are significantly decreased in the maternal circulation of women with preeclampsia (Dordevic et al., 2008). Reduced SOD activity also has been reported in placentas of women with preeclampsia, suggesting that the total antioxidant protective capacity was decreased in women with preeclampsia (Zhang et al., 2008). Decreased levels of antioxidant vitamins C, A, E, and glutathione levels were also observed in the maternal circulation of women with preeclampsia (Cindrova-Davies, 2014). And administration of vitamins C and E can block hypoxia-reoxygenation (HR)-mediated sFlt-1 secretion, via inhibition of p38 and NF-κB signaling pathways (Cindrova-Davies et al., 2007; Cindrova-Davies, 2009). Thus, antioxidant treatment may be a potential strategy for preeclampsia therapy.

Alpha-1 antitrypsin (AAT), a serine protease inhibitor, is one of the most abundant proteins in human peripheral serum, which is primarily produced by the liver, lungs, and human amniotic epithelial cells (Zsila, 2010). AAT can neutralize elastase that is required for neutrophil migration and for tissue remodeling (Al-Omari et al., 2011). Adequate inhibitory activity of AAT is critical for the prevention of proteolytic tissue damage and anti-inflammation (Catarino et al., 2012). It was reported that AAT levels was significantly increased during the third trimester of pregnancy. However, AAT levels were lower in the women with preeclampsia than the healthy women and correlated with protease inhibitory capacity (Twina et al., 2012). Cindrova-Davies et al. demonstrated that oxidative stress induced by HR could activate the p38 and stress-activated protein kinase mitogen-activated protein kinase (MAPK) pathways as well as nuclear factor-κB pathway, which mediated the release of proinflammatory factors involved in development of PE (Cindrova-Davies et al., 2007). Our previous study have demonstrated that exogenous AAT alleviated hypoxia/reoxygenation injury in a dose- and time- dependent manner, and overexpression of AAT inactivated PAK/p38 signaling and suppressed oxidative stress, indicating that AAT played a cytoprotective role in vascular endothelial cell (Feng et al., 2015). In this study, we aim to investigate whether AAT injection contributes to the improvement of PE, and to identify the mechanism behind these effects.

## Materials and methods

### Human placenta tissues and blood samples

This study was approved by Ethics Committees of Nanjing Medical University and the Wuxi Maternal and Child Health Hospital Affiliated to Nanjing Medical University. Written informed consent was obtained from all women. PE was defined as blood pressure ≥140/90 mmHg at least in two occasions, and proteinuria at least 300 mg/day on or after 20 weeks of gestation. Placenta tissue samples were obtained from 209 women postpartum: 120 normotensive pregnant and 89 preeclamptic. And blood samples were obtained from these women in 24th to 34th gestational weeks. The clinical characteristics of normotensive women and preeclamptic women were shown in Table 1. The placenta volume and weight of newborn from preeclamptic women was lower than that from normotensive women (Table 1).

**Table 1:** **Clinical characteristics of normotensive and preeclamptic women**.

**Variables**	**Normotensive (*n* = 120)**	**Preeclamptic (*n* = 89)**	***t*-test *P***
Age	28.78 ± 0.48	30.62 ± 0.64	0.85
Placenta volume (cm^3^)	61.54 ± 2.61	56.62 ± 2.15	0.015
Crown-lump length (cm)	6.14 ± 0.09	6.06 ± 0.11	0.99
Placenta quotient^#^	9.96 ± 0.29	9.13 ± 0.38	0.083
Week of gestation (week)	39.66 ± 0.09	38.92 ± 0.18	0.001
Weight of newborn (g)	3419 ± 35.97	3286 ± 53.76	0.034

# *Placenta quotient, Placenta volume/Crown-lump length*.

### PE model preparation

Institute of Cancer Research (ICR) mice were obtained from Hunan SJA Co. (Changsha, China). The experimental animals in this study were approved by the Institutional Animal Care and Research Advisory Committee of Nanjing Medical University. The female mice were randomly divided into normal pregnant group (*n* = 8) and PE model group (*n* = 32). The mice were raised with the female-male ratio of 1:1 in the same rearing cage for mating. The time after the morning of the observation of the vaginal plug was designated as post-coital day 0.5. The PE model was prepared as previously described (Omatsu et al., 2005). Briefly, phosphatidylserine/dioleoyl-phosphatidycholine (PS/PC) (Sigma-Aldrich, St. Louis, MO, USA) was prepared by mixing 80% dioleoyl-PC and 20% PS with a final concentration of 10 mg/ml. One hundred microliters (1 mg) of PS/PC suspension (PE group, 32 animals) and 100 μl of saline as a control (normal group, eight animals) were injected into tail veins every day from days 5.5 to 16.5 of pregnancy. In PE group, the mice were randomly divided into four groups each for eight animals: PE+ saline, PE+ AAT 25, PE+ AAT 50, and PE+ AAT 75. AAT solution (25, 50, or 75 mg/kg) and 100 μl of saline as a control were injected into tail veins every day from days 0.5 to 16.5 of pregnancy.

### Blood pressure, urinary protein, and placenta collection

Non-invasive rat tail blood pressure detecting method was used to measure blood pressure after the behavior and heart rate had stabilized on days 0.5, 14.5, and 16.5. The measurement was repeated at least three times. The proteinuria was measured on day 17.5. On day 17.5 of pregnancy, the animals were euthanized under the anesthesia by overdose chloral hydrate. The peripheral blood was collected for further analysis. The animals were dissected, and the fetuses were obtained to evaluate the weights and placentas were collected for western blot and IHC analysis.

### Reagents

Primary antibodies: rabbit monoclonal anti-alpha 1 antitrypsin (cat no. ab179443), rabbit monoclonal anti-PAK1 (cat no. ab40852), rabbit monoclonal anti-PAK1 (phospho S199; cat no. ab192814), rabbit monoclonal anti-PAK1 (phospho T212; cat no. ab75599), rabbit polyclonal anti-STAT1 (cat no. ab2415), mouse monoclonal anti- STAT1 (phospho Y701; cat no. ab29045), rabbit monoclonal anti- STAT1 (phospho S727; cat no. ab109461), rabbit polyclonal anti-p38 (cat no. ab7952), and rabbit monoclonal anti-p38 (phospho T180 + Y182; cat no. ab195049) from Abcam (Cambridge, UK); rabbit polyclonal anti- p38 (phospho Tyr322; cat no. AP50174) from Abgent (San Diego, CA, USA); mouse monoclonal anti-GAPDH (cat no. SC-365062) were from Santa Cruze (Dallas, Texas, USA). Horseradish peroxidase-conjugated secondary goat anti-mouse and goat anti-rabbit antibodies were from Boster (Wuhan, China). HRP-polymer anti-mouse IHC kit and HRP-polymer anti-rabbit IHC kit were from Maixin (Fuzhou, China). ELISA Kit for Alpha-1-Antitrypsin (AAT), ELISA Kit for Nitric Oxide Synthase 3, Endothelial (eNOS), ELISA Kit for Matrix Metalloproteinase 9 (MMP9), and ELISA Kit for soluble fms-like tyrosine kinase-1 (sFlt-1) were from Cloud-Clone Corp. (Houston, TX, USA); superoxide dismutase (SOD) activity assay kit and glutathione peroxidase (GPx) activity assay kit were from Biovision (Milpitas, California, USA); lipid peroxidation malondialdehyde (MDA) assay kit and reactive oxygen species (ROS) assay kit were from Beyotime (Shanghai, China).

### Immunohistochemical (IHC) staining

The placenta tissues were embedded in paraffin and cut into 4 μm slides. The slides were deparaffinized in dimethylbenzene and hydrated in alcohol gradient. After inactivating endogenous peroxidase in 3% hydrogen peroxide, slides were then retrieved in citric acid buffer (pH6.0) by microwave for 15 min. Slices were blocked by normal goat serum, and incubated with primary antibody overnight at 4°C. The slides were then washed with TBST and incubated with appropriate secondary antibody for 2 h at 37°C. The sections were then washed with TBST and stained by using DAB Detection Kit (Solarbio, Beijing, China). Finally, the sections were counterstained with hematoxylin.

### Western blot analysis

RIPA lysis buffer was used to extract protein from indicated cells. BCA Protein Assay Kit (Thermo Scientific, USA) was used to measure the protein concentration. Total 60 μg of protein were separated on 10% SDS-PAGE gels and blotted onto nitrocellulose membranes. The membranes were blocked for 2 h with 5% non-fat dry milk diluted with tris buffered saline (TBS) and incubated with primary antibodies overnight at 4°C. The membranes were washed with TBST, and then incubated with appropriate horseradish peroxidase-conjugated secondary antibody for 1 h at 37°C. Enhanced chemiluminescence reagent (Merck Millipore, Germany) was used to detect the signal on the membrane. The expression of genes was analyzed by normalizing to the expression of the internal control (GAPDH).

### ELISA

ELISA kits were used to detect the levels of AAT, eNOS, MMP9, s-Flt, ROS, MDA, SOD, and GPx in the serum according to manufacturer's instructions.

### Statistical analysis

Statistical analyses were performed using GraphPad Prism 5 software (Graphpad Software, Inc., La Jolla, CA, USA) and the data are presented as the mean ± standard deviation. An unpaired two tailed Student's *t*-test or one way analysis of variance (ANOVA) with Bonferroni *t* post-test was used to analyze the data depending on conditions. *P* < 0.05 was considered to indicate a statistically significant difference.

## Results

### AAT contributes to the development of preeclampsia

**Figure 1:**
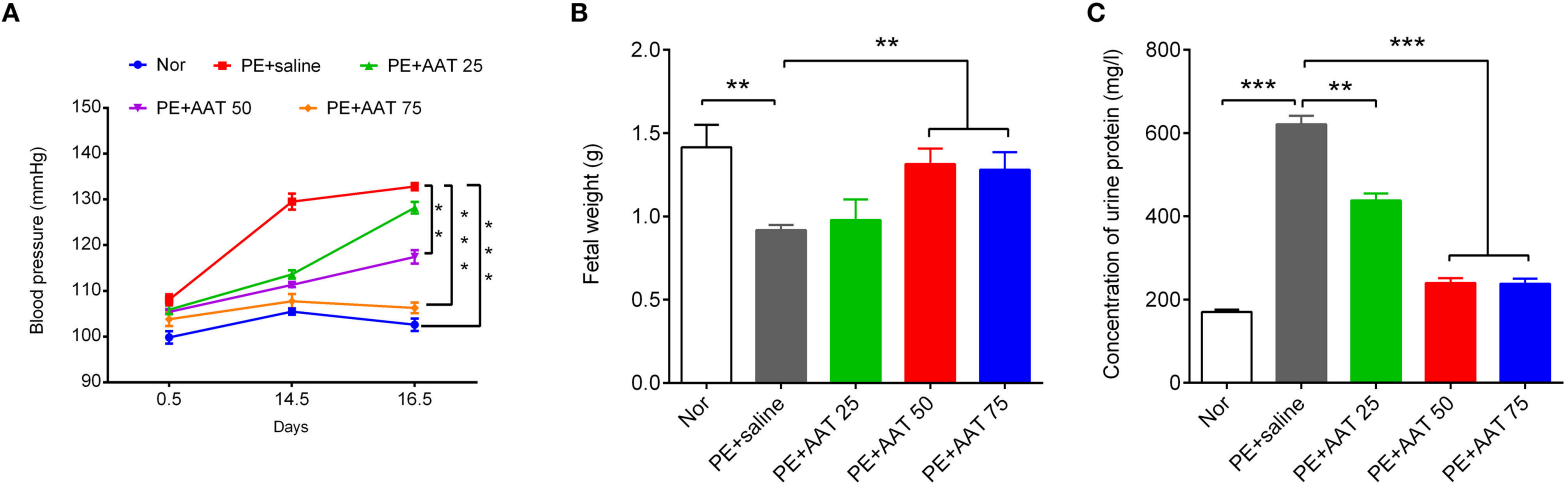
**AAT decreases blood pressure and urinary protein levels, and increases fetal weights in PE animal model**. **(A)** Non-invasive rat tail blood pressure detecting method was used to measure blood pressure on days 0.5, 14.5, and 16.5; the measurement was repeated at least three times. **(B)** The fetuses were obtained to evaluate the weights; **(C)** The proteinuria was measured on day 17.5 of pregnancy. *N* = 8. ^**^p < 0.01, ^***^p < 0.001.

**Figure 2:**
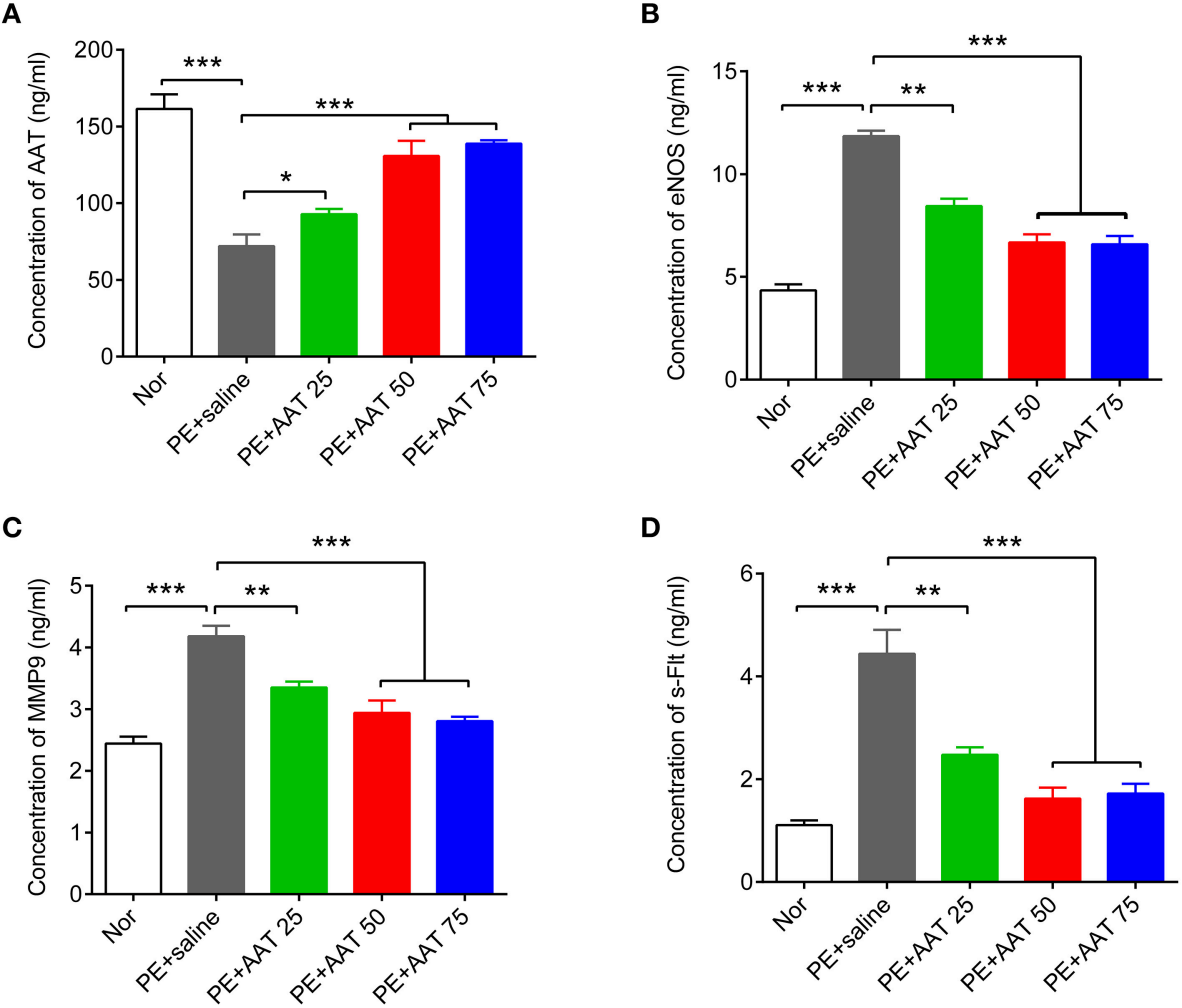
**AAT decreases the concentration of eNOS, MMP9, and s-Flt in blood from PE animals**. ELISA was used to detect the concentration of AAT **(A)**, eNOS **(B)**, MMP9 **(C)**, and s-Flt **(D)** in serum from PE or normal animals after indicated treatment. *N* = 8. ^*^p < 0.05, ^**^p < 0.01, ^***^p < 0.001.

We established PE animal model by injecting ps/pc into pregnant mice. As shown in Figure 1, the results showed that ps/pc injection significantly increased blood pressure on day 14.5, decreased the fetal weight and elevated the urine protein levels compared with the normal group, indicating that the PE model was successfully established. To investigate the role of AAT on PE, we injected a range of AAT solution (25, 50, or 75 mg/kg) into tail veins every day from days 0.5 to 16.5 of pregnancy in PE mice, and 100 μl of saline injection was used as a control. We found that AAT injection significantly relieved the high blood pressure, increased the fetal weight and reduced urine protein levels in a dose-dependent manner (Figure 1). Notably, we found that the concentration of AAT in peripheral serum was significantly lower in PE mice than normal mice (Figure 2A). Furthermore, we gradually increased concentration of AAT in peripheral serum (Figure 2A) and then analyzed some biomarkers of PE in peripheral serum. We observed the fact that the concentrations of eNOS, MMP9, and s-Flt in serum were significantly increased in PE mice (Figures 2B–D). As expected, the concentrations of eNOS, MMP9, and s-Flt in serum were reduced with increased dosage of AAT (Figures 2B–D). Thus, these results suggest that AAT injection is able to relieve the symptoms of PE mediated by ps/pc.

### AAT suppresses oxidative stress induced by PE

**Figure 3:**
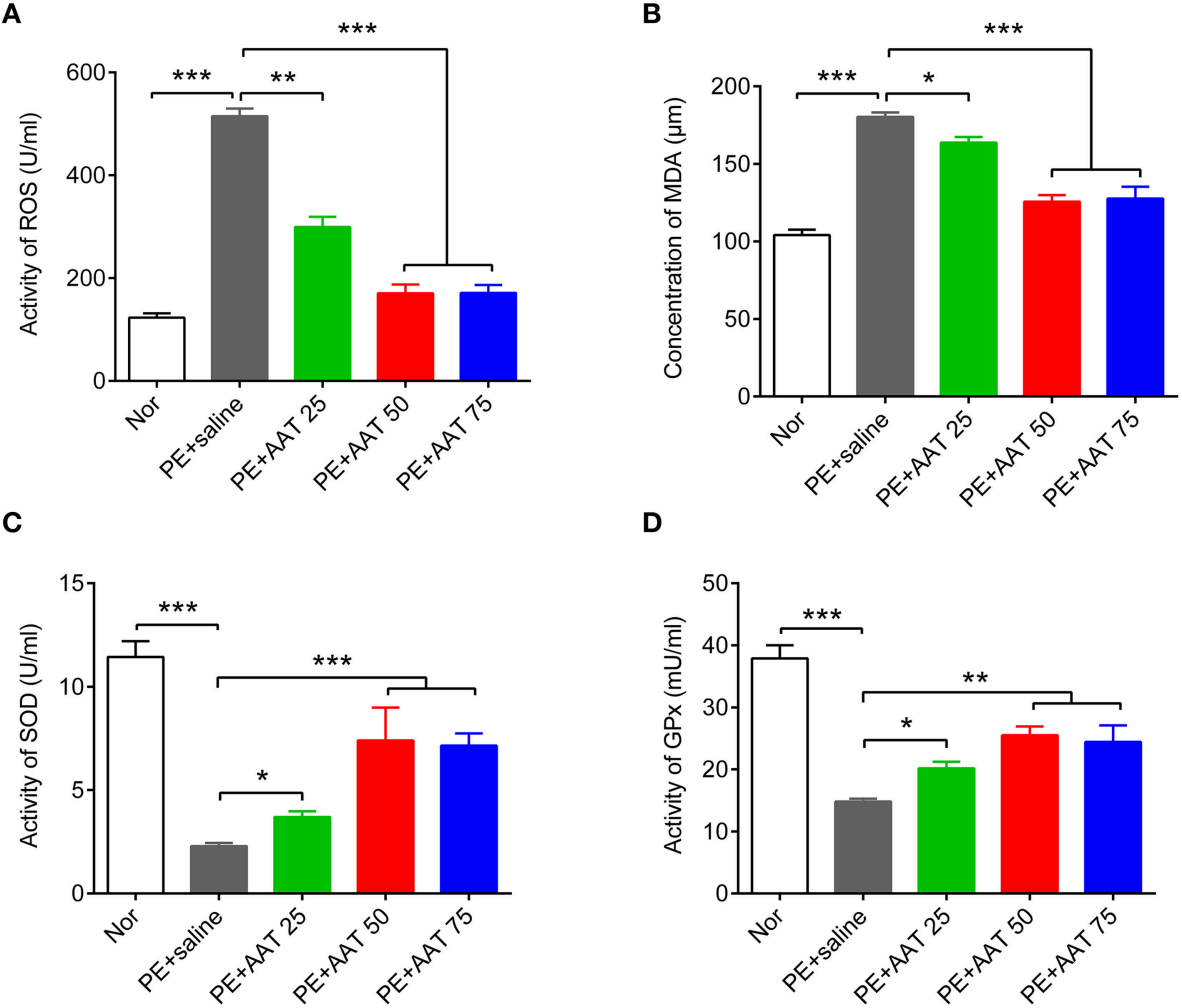
**AAT decreases the activity of ROS and the concentration of MDA, whereas increases the activity of SOD and GPx**. ELISA was used to detect the actvity of ROS **(A)**, the concentration of MDA **(B)**, and the activity of SOD **(C)**, and GPx **(D)** in serum from all groups. *N* = 8. ^*^p < 0.05, ^**^p < 0.01, ^***^p < 0.001.

Oxidative stress is an important risk factor for PE. We next investigate whether AAT can suppress oxidative stress to improve PE. We analyzed the oxidative stress related factors, including ROS, lipid peroxidation malondialdehyde (MDA), SOD, and glutathione peroxidase (GPx), in peripheral serum. Our results showed that AAT injection significantly reduced PE mediated-upregulation of ROS and MDA, while increased the levels of SOD and GPx with increased dosage of AAT (Figure 3).

### AAT inactivates PAK/STAT1/p38 signaling

**Figure 4:**
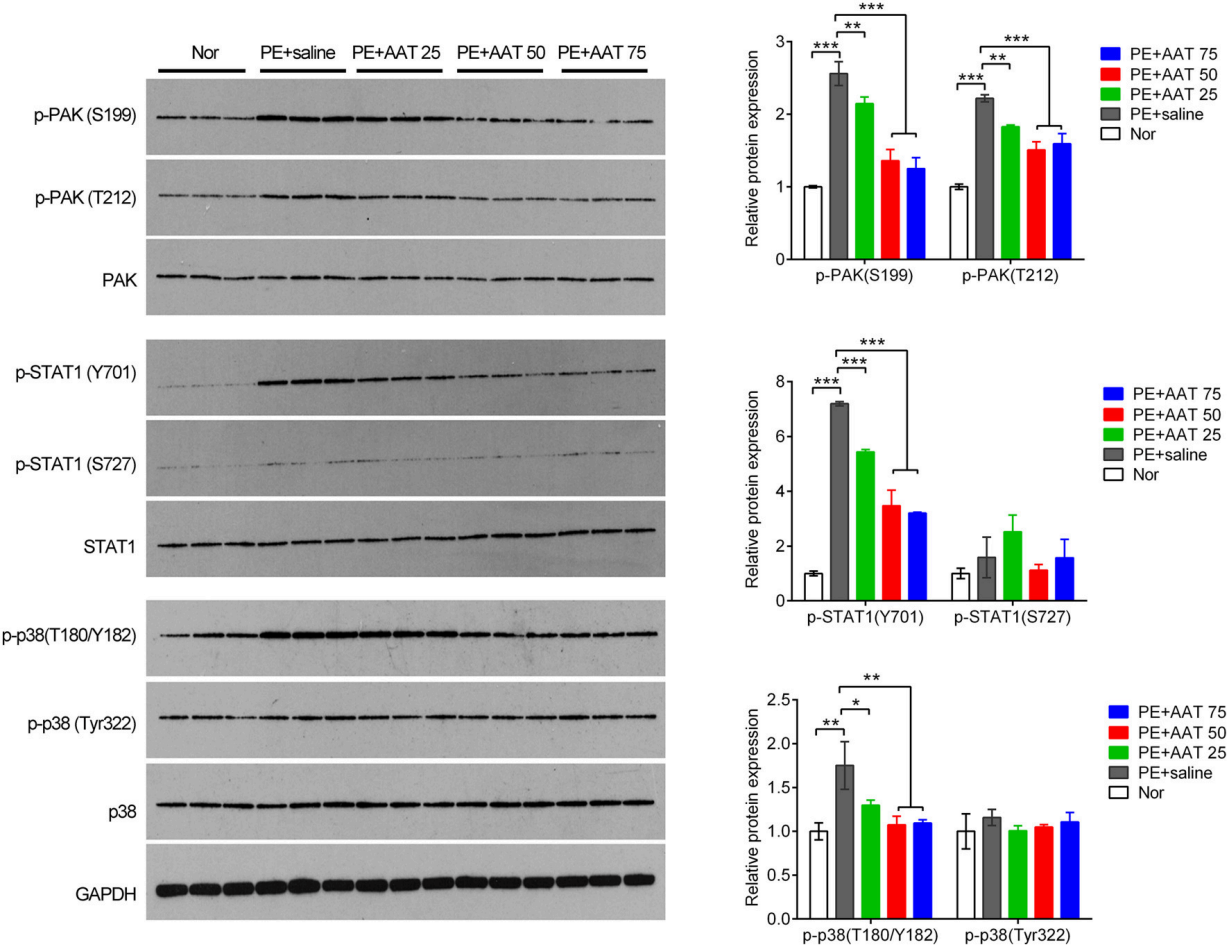
**AAT inactivates PAK/STAT1/p38 signaling that enhanced by PE**. Western blot analysis for PAK, phosphorylated PAK, STAT1, phosphorylated STAT1, p38 and phosphorylated p38 in mice placenta. *N* = 3. ^*^p < 0.05, ^**^p < 0.01, ^***^p < 0.001.

**Figure 5:**
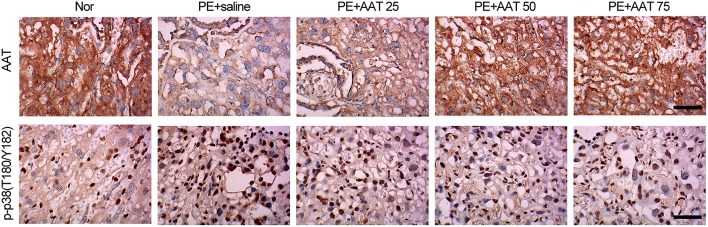
**AAT decreases the expression of phosphorylated p38 (T180/Y182) in placenta from PE mice**. Representative images of IHC staining for AAT. AAT expression was decreased in placenta from PE mice, and significantly increased after AAT injection (upper); Representative images of IHC staining for phosphorylated p38 (T180/Y182). p-p38 (T180/Y182) expression was enhanced in placenta from PE mice, and significantly decreased after AAT injection (lower). Bar = 50 μm. *N* = 8.

In addition, we obtained the placenta tissues from the pregnant mice to analyze oxidative stress related downstream molecules. We found that PE activated the PAK/STAT1/p38 signaling that enhanced the phosphorylation levels of these genes. We demonstrated that AAT was able to reduced the phosphorylation levels of PAK1 at both of S199 and T212 sites, whereas decreased the phosphorylation levels of STAT1 and p38 at Y701 and T180/Y182, respectively, but not alter the phosphorylation levels of STAT1 and p38 at S727 and Tyr322 (Figure 4). Furthermore, we performed IHC assay to confirm that upregulation of AAT was observed in placenta tissues and the phosphorylation levels of p38 at T180/Y182 was also significantly decreased by AAT injection in placenta tissues (Figure 5).

### AAT levels are lower in human placenta tissues of PE patients than that of health people

**Figure 6:**
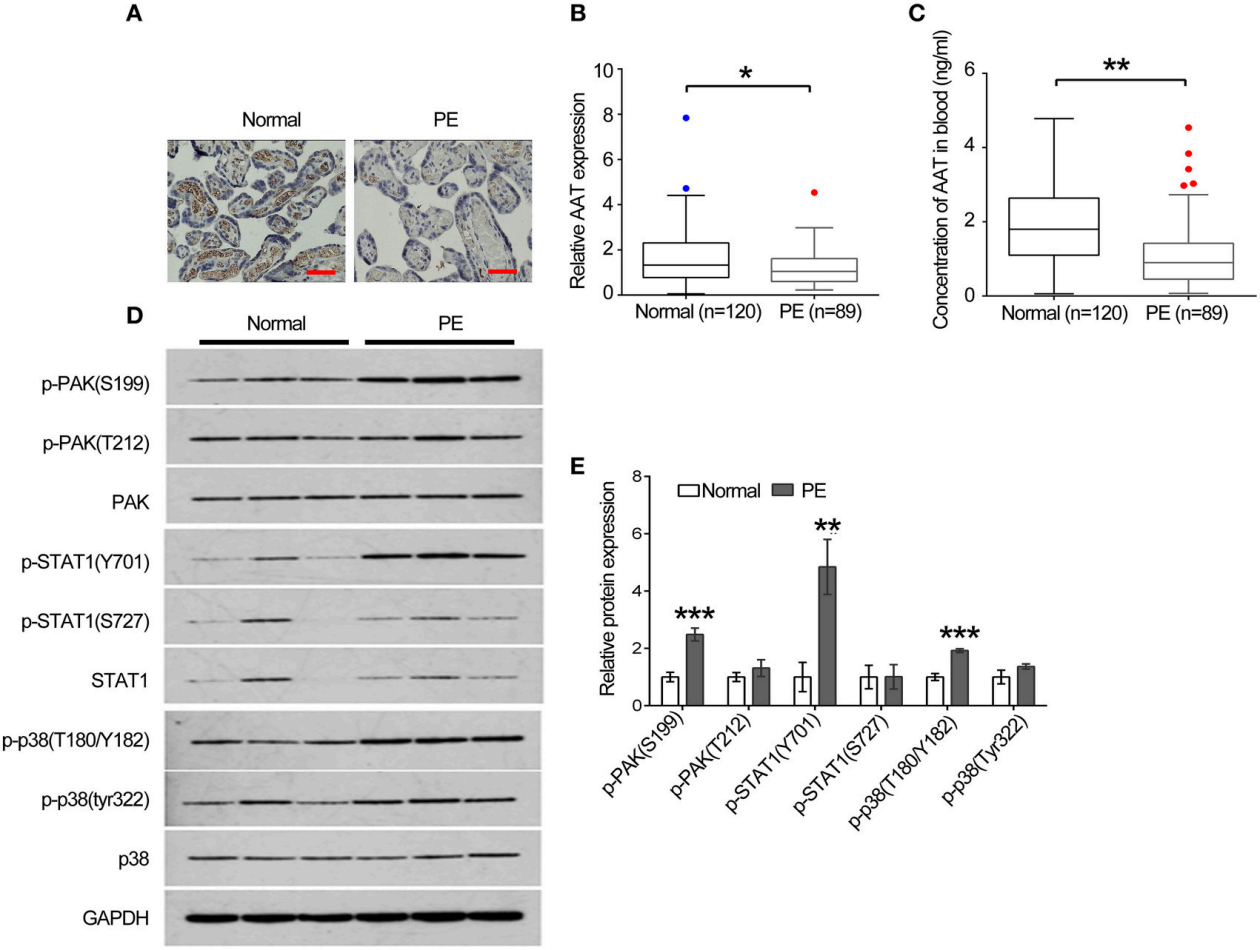
**The expression of AAT was dramatically decreased in blood and placenta tissues from PE patients**. **(A)** Representative images of IHC staining for AAT. Bar = 50 μm. **(B)** AAT expression was decreased in placenta from PE patients. **(C)** ELISA was used to detect the concentration of AAT in blood from PE patients. **(D)** Western blot analysis for PAK, phosphorylated PAK, STAT1, phosphorylated STAT1, p38 and phosphorylated p38 in human placenta. **(E)** Quantification of the band in **(C)**. ^*^p < 0.05, ^**^p < 0.01, ^***^p < 0.001.

To further confirm our results in animals, we collected 120 placenta samples from health people and 89 placenta samples from PE patients to perform IHC to analyze the expression of AAT. Clinically, our data reveal that AAT levels were lower in placenta tissues of PE patients than that of in health people (Figures 6A,B). In addition, we also detected the concentration of AAT in blood from the health people and PE patients using ELISA. In line with the results shown in IHC, the concentration of AAT (1.08 ± 0.08 ng/ml) in blood from PE patients was significantly decreased compared with the health people (1.9 ± 0.18 ng/ml; Figure 6C). Furthermore, we analyzed the key molecules in PAK/STAT1/p38 signaling in human placenta samples using western blot. We found that the expression levels of p-PAK1(S199), p-STAT1(Y701), and p-p38(T180/Y182) was significantly increased in placenta tissues of PE patients compared with that of in health people (Figures 6D,E).

## Discussion

In this study, we find that AAT levels are significantly decreased in placenta tissues from women with PE compared that of healthy women. Notably, we demonstrate that AAT injection is able to relieve the high blood pressure and reduce urine protein levels in a dose-dependent manner, and thus improve PE.

AAT is a typical serine protease inhibitor with potent anti-neutrophil protease activities, such as against elastase and proteinases, which plays an important role in controlling tissue damage by proteases in the microenvironment of inflammation (Bironaite et al., 2001). Significant decreases in serum levels of human AAT have been associated with the development of emphysema. The decreased levels or activity of AAT are attributed in part to excessive polymerization of the mutant protein and to post-translational changes induced by oxidative stress. And AAT levels were lower in the woman with preeclampsia than the healthy woman (Twina et al., 2012). It seems conceivable that polymerized serine protease inhibitor may occur in hepatocytes (Mahadeva et al., 1999), contributing to low levels of circulating AAT in woman with preeclampsia.

AAT also exhibits a procellular survival effect in models of serum deprivation and ischemia-reperfusion injury in vascular endothelial cells and in alveolar endothelial cells via direct caspase-3 inhibition (Petrache et al., 2006). Smoking can enhance oxidative stress. Increased lipid peroxide and nitric oxide end products, and decreased superoxide dismutase (SOD) activity and serum AAT were observed in cigarette smokers, and further increase in number of cigarettes per day exacerbates the oxidative stress with decrease in AAT (Sayyed et al., 2008; Wada et al., 2012), which may serve as a marker of oxidative stress (Ueda et al., 2002). Children with alpha-1 antitrypsin deficiency showed increased oxidative stress in the serum and have significantly lower total glutathione and reduced glutathione levels leading to an accumulation of hydrogen peroxide that would explain the significantly increased levels of oxidative stress biomarkers observed in these patients (Escribano et al., 2015). Our results showed that AAT injection exhibited an anti-oxidative stress role by significantly reducing PE mediated-upregulation of ROS and MDA, and increasing the levels of SOD and GPx with increased dosage of AAT. In addition, AAT injection also increased the expression of endothelial nitric oxide synthase (eNOS) but decreased the expression of matrix metalloproteinase 9 (MMP9), a useful biomarker of susceptibility to severe PE and its early onset related to insufficient invasion of trophoblast leading to superficial and unsuccessful placentation (Shokry et al., 2009; Wang et al., 2010). Lockwood CJ showed that tumor necrosis factor-α (TNF-α) significantly enhanced MMP-9 mRNA and protein levels and activity, suggesting that excess macrophage-derived TNF-α augmented expression of MMP-9 in decidual cells to interfere with normal stepwise extravillous trophoblast invasion of the decidua. They also showed that p38 mitogen-activated protein kinase signaling mediated TNF-α enhancement of MMP-9(Lockwood et al., 2014). Thus, activation of p38 signaling might indirectly regulate MM9 expression and activity via increasing the release of proinflammatory cytokines. Increased circulating levels of anti-angiogenic factors, such as soluble fms-related tyrosine kinase-1 (sFlt-1), were observed in PE (Cindrova-Davies, 2014). And it was reported that p38 inhibition provides protective effects in H/R-exposed human umbilical vein endothelial cells (HUVECs) by suppressing oxidative stress, inhibiting apoptosis, and promoting their potential for *in vitro* angiogenesis; H/R intervention causes the induction of Gadd45α leading to p38 activation and ultimately an increase in sFlt-1 secretion in HUVECs (Luo et al., 2011). These studied indicate that the anti-angiogenic factors, such as soluble fms-related tyrosine kinase-1 (sFlt-1), as well as proinflammatory cytokines, such as TNF-α and IL-1β, may be the potential downstream effectors of PAK/STAT1/p38 signaling pathway in PE. Nitric oxide (NO), one of the major endothelium-derived vasoactive mediators, is synthesized by endothelial NO synthases (eNOS). An increased eNOS expression and hence increased NO production may be an adaptive response to the increased resistance and poor perfusion in the pathological pregnancies (Myatt et al., 1997). SOD is an antioxidant enzyme highly specific for superoxide elimination. An increased oxidative level and decreased antioxidant activities in the peripheral blood of women with preeclampsia have been reported (Suhail et al., 2009), suggesting that oxidative stress markers play a significant role in the pathophysiology of pre-eclampsia (Sharma et al., 2006). Inadequate amount of antioxidant enzymes in circulation may be important contributing factor associated with oxidative stress leading to endothelial dysfunction in mothers with preeclampsia (Negi et al., 2012). The levels of GPx, SOD and MDA were significantly higher in women with PE than in healthy women, and the increase was higher in women with severe PE (Bulgan et al., 2005; Chamy et al., 2006). Reduced GPx levels could be associated with increased generation of toxic lipid peroxides contributing to the endothelial dysfunction and hypertension of preeclampsia (Krishna and Venkataramana, 2007; Mistry et al., 2008). Thus, these data suggest that upregulation of AAT is able to protect vascular endothelial cells in placenta from oxidative stress.

In addition, we find that AAT injection inactivated PE mediated activation of PAK/STAT1/p38 signaling. Our previous study showed that overexpression of AAT decreased cell apoptosis and promoted proliferation via inhibiting Rac1/PAK/p38 signaling and against oxidative stress (Feng et al., 2015). RAC1 primarily activates p21-activated kinases (PAK) and also interacts directly with p38 pathway to regulate proliferation, and regulates ROS-mediated cell killing. ROS activates multiple signaling pathway including MAPK, JAK/STAT1. Kim HS and Lee MS suggested that STAT1 signaling amplifies the initial ROS response through p38 MAPK activation in a positive-feedback mechanism (Kim and Lee, 2005). Our findings suggest that STAT1 activation by tyrosine phosphorylation but not by serine phosphorylation and subsequent p38 activation by threonine phosphorylation is responsible for PE. The importance of STAT1 phosphorylation at tyrosine in apoptosis is consistent with a previous report demonstrating the critical role of tyrosine phosphorylation of STAT1 in apoptosis and cytotoxicity induced by oxidative stress. Oxidative stress induces the release of proinflammatory cytokines, such as TNF-α, IL-1β, and COX-2, while IL-1β treatment increased p38 activity (Szabo et al., 2015). In turn, p38 pathway has been implicated in the posttranscriptional regulation of TNF-α and IL-1. Inhibition of the p38 MAPK pathway significantly suppressed H/R-induced COX-2 expression (Cindrova-Davies et al., 2007). There is therefore the possibility of developing an autocrine feed-forward system (Cindrova-Davies et al., 2007). Our results showed that AAT injection exhibited an anti-oxidative stress role by significantly reducing PE mediated-upregulation of ROS and MDA, and increasing the levels of SOD and GPx with increased dosage of AAT. It is possible that AAT protects vascular endothelial cells in placenta by inhibition oxidative stress, and subsequent decrease in release of proinflammatory cytokines, resulting in inactivation of p38 signaling. Oxidative stress induces the placenta to release various factors, including inflammatory cytokines and antiangiogenic factors, resulting in an enhanced endothelial dysfunction, which may happen at early stage of pregnancy. However, there is no reduction in the incidence of the disease after treating women at risk of preeclampsia with antioxidant (Poston et al., 2006; Rumbold et al., 2006). Considering the close relationship between oxidative stress and p38 signaling, we infer that p38 activation may occur at early stage of pregnancy or at more early stage to promote oxidative stress and mediate PE, which could not reverse already established disease pathology at late stage. Despite key roles of STAT1/p38 in PE induced by ps/pc injection at present, we cannot rule out the involvement of another signaling molecule such as β-catenin signaling. Overexpression of C/EBPβ might influence the activity of MMPs by regulating the Wnt/β-catenin signaling pathway to affect the invasion of trophoblast cells, which then participate in the pathogenesis of preeclampsia (Zhuang et al., 2015). Taken together, our study suggests that exogenous AAT improves PE via inactivating STAT1/p38 signaling.

In conclusion, our study suggests that exogenous AAT injection increases the antioxidants and suppresses oxidative stress, and subsequent improvement of PE through inactivation of STAT1/p38 signaling. Thus, AAT would become a potential strategy for PE therapy.

## Author contributions

Conceived and designed the study and experiments: JJ and QZ. Patients were selected by: YL and JJ. Performed the experiments: YL, RW, NL, YQ, HY, and HS. Analyzed and discussed the data and discussed the written manuscript: All authors. Wrote the manuscript: YL, JJ, and QZ. Constructed the figures and tables: YL, HS, JJ, and QZ.

## Funding

This work was supported by Jiangsu provincial science and Technology Department of the key disease standardized diagnosis and treatment project (No.BE2015618), standardized diagnosis and treatment projects research program of Medical Science and Technology Development Fund of the Medical Control Center in Wuxi City (No.YGZXG1408), the Wuxi Bureau of Science and Technology Medical Technology Development Fund (No.CSE31N1321), and Maternal and Child Health research project in Jiangsu province (F201422).

### Conflict of interest statement

The authors declare that the research was conducted in the absence of any commercial or financial relationships that could be construed as a potential conflict of interest.
